# Humidity-Induced Phase Transitions of Surfactants Embedded in Latex Coatings Can Drastically Alter Their Water Barrier and Mechanical Properties

**DOI:** 10.3390/polym10030284

**Published:** 2018-03-08

**Authors:** Juan F. Gonzalez-Martinez, Yana Znamenskaya Falk, Sebastian Björklund, Stefan Erkselius, Nicola Rehnberg, Javier Sotres

**Affiliations:** 1 Biomedical Science Department & Biofilms-Research Center for Biointerfaces, Malmö University, 20506 Malmö, Sweden; juan.fransisco.gonzales@mau.se (J.F.G.-M.); yana.znamenskaya@mau.se (Y.Z.F.); sebastian.bjorklund@mau.se (S.B.); 2 Bona AB, 20021 Malmö, Sweden; stefan.erkselius@bona.com (S.E.); nicola.rehnberg@bona.com (N.R.)

**Keywords:** latex, surfactant, coating, relative humidity, QCM-D, AFM

## Abstract

Latex coatings are environmentally friendly i.e., they are formed from aqueous polymer dispersions, are cheap to produce and provide exceptional mechanical properties. Therefore, they are ubiquitous and can be found in a wide range of different applications such as paints and varnishes, pressure-sensitive adhesives, textiles, construction materials, paper coatings and inks. However, they also have weaknesses and their surfactant content is among them. Surfactants are often needed to stabilize polymer particles in the aqueous latex dispersions. These surfactants also form part of the coatings formed from these dispersions, and it is well-known that they can lower their performance. This work further explores this aspect and focuses on the role that embedded surfactant domains play in the response of latex coatings to humid environments. For this purpose, we made use of several experimental techniques where humidity control was implemented: quartz crystal microbalance with dissipation, atomic force microscopy and differential scanning calorimetry. By means of this multimethodological approach, we report that surfactants embedded in latex coatings can undergo humidity-induced transitions towards more hydrated and softer phases, and that this results in a drastic decrease of the mechanical and water barrier properties of the whole coatings. Subsequently, this work highlights the potential of taking into account the phase behavior of surfactants when choosing which ones to use in the synthesis of latex dispersions as this would help in predicting their performance under different environmental conditions.

## 1. Introduction

Driven by environmental legislation worldwide and by their improving performance, the use of waterborne coatings is continuously increasing to the detriment of solvent-borne coatings [1]. In many cases, waterborne coatings result from casting on a surface an aqueous dispersion of (mostly) hydrophobic polymer particles (i.e., latices). A common way to stabilize these dispersions is by using surfactants which adsorb on the surface of the particles. Surfactants also aid in other aspects such as wetting, foaming control and prevention of film defects caused by surface tension gradients [2]. However, surfactants are not volatile and after casting of the dispersion and the subsequent evaporation of water they are incorporated on the coatings. It has been extensively reported that the presence of surfactants in latex coatings has a strong influence on their performance. The specific influence is highly determined by their distribution within the coating [1]. Therefore, there has been enormous efforts in determining this distribution [1,3,4,5,6]. This have been proved a complex problem as it exhibits dependence on the specific characteristics and relative concentrations of surfactants and polymers, drying conditions, etc. [1].

It is also known that most surfactants, especially those of ionic character, undergo phase segregation with the latex polymer, forming surfactant domains in the bulk of the film or migrating to its surfaces [1,7]. Nonuniform surfactant distributions affect film properties in many ways [1]. The influence on appearance is well-described [8,9,10]. The presence of surfactant domains in cured coatings also plays a critical role in their water resistance and mechanical properties. Their presence in the interior of the coating is a source of mechanical weakness [11], while their presence at the substrate/coating interface adversely affects adhesion [12]. Water resistance is also adversely affected by the presence of surfactant domains [13,14]. Moreover, both aspects are interconnected as both water and surfactants can contribute with plasticization effects [15,16,17]. However, while the effects of exposing latex coatings to liquid water have been extensively studied, this is not the case for the exposure to humid environments.

This work focuses on the influence of humidity on latex coatings. It was driven by initial observations that indicated that humidity has a significant influence on the hardness of the coatings. For this study, we used several experimental techniques, where humidity-control was implemented, to investigate a model latex coating and the surfactant used in the polymerization process of the corresponding latex dispersion. The reported experimental results provide a solid mechanistic understanding on the critical role that embedded surfactant domains have on the response of the coatings to variations in the ambient humidity.

## 2. Materials and Methods

### 2.1. Latex Coatings

The investigated latex coatings were prepared by further diluting a commercial latex dispersion in a film forming aqueous solution. Specifically, the original latex dispersion (Polymer HD7701, kindly provided by BW Resins AB, Malmö, Sweden) had a solid content of 41% *w*/*w* consisting of methyl methacrylate-*co*-butyl acrylate-*co*-methacrylic acid (MM-*co*-BA-*co*-MAA) particles (diameter ca. 110 nm, glass transition temperature, *T*_g_, 18 °C) stabilized by the anionic surfactant Rhodacal^®^ DSB (Sodium Alkyl Diphenyl Oxide Sulfonate, Solvay Novecare, Oldbury, UK). Before use, the *Polymer HD7701* dispersion was diluted in a 2/3 ratio in the film forming solution mentioned above, which contained 24.7% *w*/*w* of dipropylene glycol methyl ether (DPM, Brenntag Nordic AB, Malmö, Sweden), 0.6% *w*/*w* of Capstone FS63 (DuPont Sverige AB, Malmö, Sweden), 5.8% *w*/*w* of BYK-093 defoamer (BYK-Chemie GmbH, Wesel, Germany) and 68.9% *w*/*w* water content.

### 2.2. Pendulum Hardness

A commercial König pendulum (Standard ISO 1522, BYK-Gardner GmbH, Geretsried, Germany) was used to characterize the hardness of the latex coatings. This method evaluates hardness by measuring the number of oscillations that it takes for an oscillating pendulum, which rests with two stainless steel balls on the coated surface to dampen. For these experiments, the original latex dispersion was applied on glass plates by using a standard 120 µm applicator and then allowed to dry at 23 °C and 50% RH (relative humidity) for one week. After one week, half of the samples were stored at 100% RH while the other half was kept at 50% RH (temperature was kept constant at 23 °C in both cases). Samples were then stored in these conditions for 7 and 14 days. After these equilibration periods, the samples were immediately (<1 min) characterized by means of the König pendulum and their hardness provided in terms of number of oscillations. For each preconditioning RH and time period, a mean and standard deviation hardness value was calculated from the measurements on two different coatings.

### 2.3. Quartz Crystal Microbalance with Dissipation (QCM-D)

Quartz crystal microbalance with dissipation (QCM-D) measurements were performed by using an E4 system (Q-Sense AB, Gothenburg, Sweden). A detailed description of the technique and its basic principles can be found elsewhere [18]. Briefly, an alternating-current voltage is applied through a gold-coated quartz chip to stimulate the shear mode oscillation of the quartz crystal. Specifically, in our experiments we used sensors (model QSX303, Q-sense AB, Gothenburg, Sweden) where the gold electrodes are covered by a thin silica layer. Adsorption of a certain amount of mass onto the sensor surface leads to a decrease in the frequency of the resonance overtones, *f_n_*. Along with the shifts in *f_n_*, QCM-D is able to detect changes in the dissipation factor, D*_n_*, of each of the overtones [19]. The dissipation factor represents the ratio between the energy dissipated by the sensor during a single oscillation after switching off the driving voltage, and the initial oscillation energy of the sensor. For the control of relative humidity (RH), the QCM-D setup was equipped with a humidity module QHM 401 (Q-Sense AB, Gothenburg, Sweden). Briefly, this consists of a chamber where a Gore membrane separates a flowing solution from the sensor. The membrane is permeable only to water vapor, which diffuses from the solution into the gas phase above the sensor and thus regulates the RH above the sensor.

Only new sensors were used for the reported experiments. Before every experiment, sensors were rinsed extensively with Ultra High Quality (UHQ) water, treated in a Hellmanex II 2% (*v*/*v*) water solution for 20 min. and subsequently rinsed again extensively with UHQ water. Finally, before being used, the sensors were dried under nitrogen and plasma-cleaned for 5 min in low pressure residual air using a glow discharge unit (PDC-32 G, Harrick Scientific Corp., Ossining, NY, USA). In a typical experiment, the clean sensor was initially characterized by means of QCM-D under dry (N_2_) atmosphere at 25 °C (RH ≈ 0%). Then, the sensor was removed from the QCM-D and subsequently a coating was formed on the sensor by spin-coating (T 25C, RH 30%, 1200 rpm, spin-coater developed in-house) 20 µL of the original latex dispersion. Immediately afterwards, the sensor was placed in the QCM-D humidity module. Then, film formation process was first monitored, and afterwards the water sorption isotherms of the coating was evaluated. The latter was performed according to the procedure described in detail elsewhere [20,21]. In brief, the method is based on the controlled dilution of a LiCl solution to adjust the water activity a_w_ in order to continuously regulate the RH (a_w_ = RH/100%) in the QCM-D chamber. In the beginning of water sorption experiments, the coatings were exposed to a dry (N_2_) atmosphere (RH ≈ 0%) until a stable baseline was observed both for the frequency and dissipation signals. This was considered as the reference dried coating. Then, an initially saturated, but continuously diluted, aqueous LiCl solution was pumped through the QCM-D humidity module where water vapor can pass across the Gore membrane and thereby set the RH of the gas phase above the coated sensor in a continuous manner. The variation of LiCl concentration with time was calculated as described in [20,21]. The water activity corresponding to the LiCl concentrations was then calculated by using published experimental relationships [22]. Control experiments were performed to confirm that the kinetics of the hydration process was independent of the water activity scan rate by changing the flow rate of the solution that dilutes the LiCl solution. In this manner, the time period for the hydration process was varied from hours to days. From these experiments, it was concluded that the hydration of the coatings was kinetically independent of the water activity scan rate, within the limits investigated. Unless otherwise stated, a pump speed of 0.1 mL/min was used. The starting volume of the saturated LiCl solution was approximately 20 mL.

#### Data Analysis

QCM-D frequency shifts for homogenously distributed and rigid films can be modelled by means of the Sauerbrey equation [23]:

(1) $$\frac{\Delta f_{n}}{n}=-\frac{2mf_{0}^{2}}{Z_{q}}$$

where *n* is the overtone number, Δ*f_n_* the frequency shift of the *n*th overtone, m is the areal mass, *f*_0_ is the quartz fundamental frequency and *Z_q_* its acoustic or mechanical impedance. It can be noted that within the Sauerbrey approximation, a similar mass would be obtained from the analysis of all overtones. However, this was not the case for the latex coatings investigated in this work. Instead, it was more accurate to use the model for single viscoelastic films in air [24]:

(2) $$\frac{\Delta f_{n}}{n}=-\frac{2f_{0}^{2}m_{f}}{Z_{q}}\left[1+\frac{Z_{q}^{2}}{3Z_{f}^{2}}{\left(\frac{m_{f}}{m_{q}}n\pi \right)}^{2}\right]$$

where *Z_f_* = (*ρ*(*G*′ + *G*″))^1/2^ is the acoustic impedance of the film, and *m_f_* and *m_q_* the areal masses of the film and of the quartz respectively. *G*′ and *G*″ are the storage and loss moduli of the film [25]. From Equation (2) it follow that for a viscoelastic film in air Δ*f_n_*/*n* has a linear dependency with *n*^2^. Thus by linear fitting Δ*f_n_*/*n* vs. *n*^2^, and by extrapolating to *n* = 0 it is possible to determine *m_f_* [25]. In addition, we have previously shown [25] that the ratio *G*′/*G*″, which is often used to describe the viscoelastic character of the films, can be approximated by:

(3) $$\frac{G\prime }{G\prime \prime }\approx \frac{-\Delta f_{n}+n{\left(\frac{\Delta f_{n}}{n}\right)}_{n\to 0}}{{\Delta \Gamma }_{n}}$$

where (Δ*f_n_*/*n*)*_n_*_→0_ is the value obtaining by extrapolating the linear fit of Δ*f_n_*/*n* vs. *n*^2^ to *n* = 0 and Γ*_n_* = *D_n_f_n_*/2 is the half-band-half-width of the *n*th overtone.

### 2.4. Differential Scanning Calorimetry (DSC)

Differential scanning calorimetry (DSC 1, Mettler Toledo, Greifensee, Switzerland) was used to characterize the thermal phase behavior of Rhodacal^®^ DSB equilibrated at different relative humidity (RH). Specifically, a liquid sample of the commercial Rhodacal^®^ DSB was loaded in standard aluminum crucibles (40 μL, Mettler Toledo, Greifensee, Switzerland) and then dried in a desiccator with 3Å molecular sieves (0% RH) for 24 h, followed by further drying in the same desiccator in vacuum at room temperature for several hours. Afterwards, completely dried Rhodacal^®^ DSB samples were equilibrated with saturated salt solutions, which give defined relative humidity levels [26], in desiccators for 5–7 days at 25 °C. Then, the desiccator with samples was placed into a glovebox, where RH was controlled by means of saturated salt solutions. Finally, samples were hermetically sealed within the glovebox and studied immediately after preparation. The RH in the desiccators and glovebox was controlled using the following saturated salt solutions: MgCl_2_ (32% RH), Mg(NO_3_)_2_ (53% RH), NaCl (75% RH), K_2_SO_4_ (97% RH) [26].

Calibration for heat flow and temperature was done using indium (m. p. 156.6 °C; ∆H = 28.45 J/g). An empty sealed pan was used as a reference during all experiments. Samples were cooled down to −80 °C, held 10 min at this temperature and then heated to 80 °C with a scan rate of 10 °C/min. Two consecutive scans were performed for all samples. The furnace chamber was purged with dry nitrogen gas flowing at 80 mL/min. Sample masses before and after DSC experiments remained the same.

### 2.5. Atomic Force Microscopy (AFM)

A commercial atomic force microscope equipped with a sealed liquid cell (MultiMode 8 SPM with a NanoScope V control unit; Bruker AXS, Santa Barbara, CA, USA) was employed to visualize the latex coatings. AFM imaging was performed at room temperature (~25 °C), by operating in the PeakForce Tapping^®^ mode. Rectangular silicon nitride cantilever with nominal spring constant 0.38 N/m (ORC8-W, Bruker AXS, Santa Barbara, CA, USA) were used. Relative humidity was controlled by flowing humid air generated with a GenRH-A setup (Surface Measurement Systems, Pragolab, Prague, Czech Republic). Analysis and processing of AFM images was performed with the WSxM software [27] and with self-programmed routines written in MATLAB (Math Works Inc., Natick, MA, USA).

## 3. Results

### 3.1. Pendulum Hardness

The König pendulum test was used to determine the hardness of the investigated latex coatings formed at 50% RH for 1 week, and then equilibrated at both 50% RH and 100% RH for 7 and 14 days. After this conditioning period, the hardness of the coatings was immediately (>1 min) determined at ambient RH (ca. 50%) (Table 1). A clear tendency was observed where lower equilibration RH led to higher hardness.

### 3.2. Quartz Crystal Microbalance with Dissipation (QCM-D)

#### 3.2.1. Latex Film Formation

**Figure 1:**
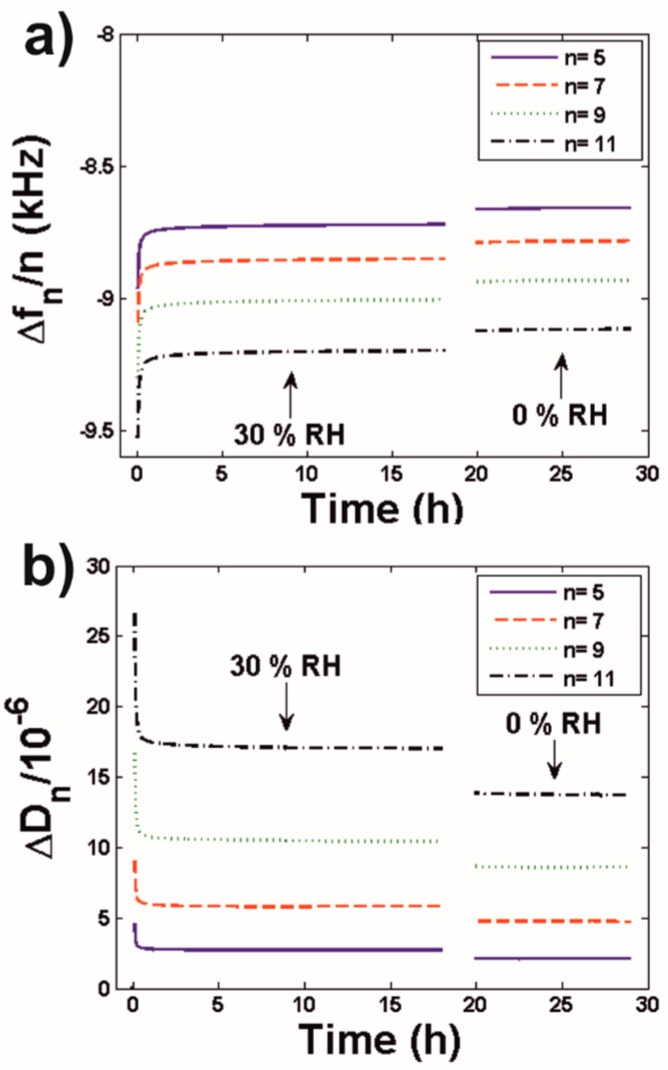
(**a**) Frequency and (**b**) dissipation shifts monitored during the film formation process of the investigated latex dispersion.

After spin-coating the latex dispersion on QCM-D SiO_2_ sensors, the sensors were immediately placed in the QCM-D system equipped with a humidity chamber and the film formation process was followed in terms of frequency and dissipation shifts. Figure 1 shows a representative example where the process was followed at 30% RH during ca. 20 h. Figure 1 also shows a subsequent characterization for 10 h at 0% RH that ensures the complete drying of the coating.

It can be observed that most of the shifts in the frequency and dissipation signals, corresponding to the evaporation of bulk water, occurred during the first 2 h of the drying process (Supplementary Material Section S1). After this period both signals stabilized. After changing RH from 30% to 0% both Δ*f_n_* and Δ*D_n_* changed in accordance with a decrease of the mass and of the viscous character of the film (i.e., less negative Δ*f_n_* values and lower Δ*D_n_* values). However, the dependence with time of both signals remained unaltered. This indicates that the loss of mass was related to the release of water absorbed within the coating i.e., the amount of water absorbed by the coatings was a function of RH, a process that also increased its rigidity (as shown by the decrease in Δ*D_n_*).

**Figure 2:**
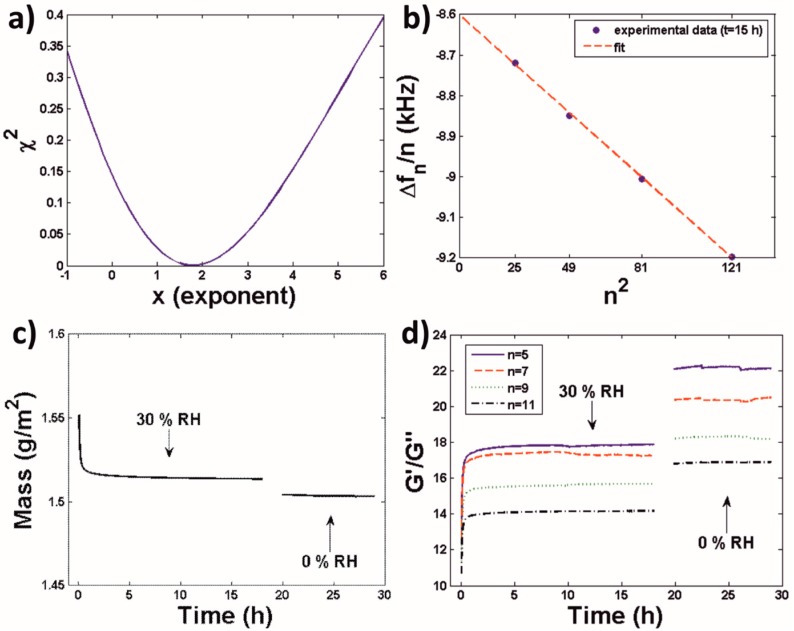
(**a**) Chi-square corresponding to the linear fit of the experimental data from Figure 1a at *t* = 15 h to the expression Δ*f_n_*/*n* = a + b*n^x^* for different *x* values. (**b**) Specific plot for Δ*f_n_*/*n* = a + b*n*^2^. (**c**) Calculated coated mass from the data in Figure 1a by using an exponent *x* = 2. (**d**) Calculated *G*′/*G*″ from the data in Figure 1a,b by using an exponent *x* = 2.

From Figure 1 is clear that Δ*f_n_* has a dependency with the overtone number, *n*. Thus, the investigated coatings could not be modelled by means of the Sauerbrey equation (Equation (1)). However, their behavior could be described by the model for viscoelastic films in air (Equation (2)). Within this model, Δ*f_n_*/*n* should exhibit a linear dependence with *n*^2^. Indeed this was the case. In Figure 2a, the goodness of linear fits of Δ*f_n_*/*n* vs. *n^x^* for −1 < *x* < 6 is shown in terms of their chi-square value Χ^2^ = Σ(observed − expected)^2^/expected, and a minimum close to *n* = 2 is observed as predicted by the model. A plot of experimental Δ*f_n_*/*n* values and the corresponding linear fit for a random time point (15 h) is shown in Figure 2b. Similar fits were obtained when evaluating the data for other time points of the film formation process (excluding the initial 2 h). This validates the use of the viscoelastic film model (Equations (2) and (3)) to estimate the mass (Figure 2c) and the *G*′/*G*″ ratio (Figure 2d) of the coatings (ΔΓ*_n_* values calculated from the frequency and dissipation shifts of Figure 1 and used in the calculation of the *G*′/*G*″ ratio are shown in Supplementary Material Section S2).

#### 3.2.2. Water Sorption Isotherms of Latex Coatings

**Figure 3:**
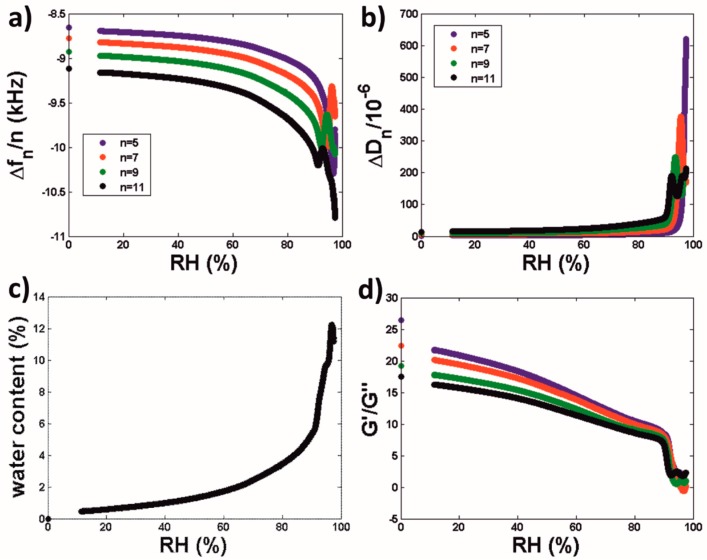
(**a**) Frequency and (**b**) dissipation shifts corresponding to a representative water sorption isotherm for a cured coating formed by spin-coating the investigated latex dispersion on a QCM-D SiO_2_ sensor. (**c**) Corresponding calculated water content and (**d**) *G*′/*G*″ values.

After exposing the cured latex coatings to a N_2_ atmosphere i.e., RH 0%, for ca. 10 h in order to completely dry them, their water sorption isotherms were monitored by means of QCM-D by exposing them to continuously increasing RH values (representative example shown in Figure 3).

When increasing the RH from 0%, the absolute frequency and dissipation shifts increased as well in a smooth way (Figure 3a,b). This behavior was observed until RH reached a value of ca. 90%. By applying Equations (2) and (3) to model this data it follows that this implies an increase the water content and viscous character of the coating (Figure 3c,d). Specifically, the water content of the coatings (Figure 3c) was calculated by calculating the coating mass for each RH according to Equation (2), and then using the relationship:

(4) $$water\;content\;\left(\%\right)=\frac{mass\;\left(RH\right)-mass\;\left(RH=0\right)}{mass\;\left(RH\right)}\;\times 100=\frac{mass_{water}}{mass_{total}}\times 100$$

At RH 90% both the frequency and dissipation shifts exhibited a peak/trough feature followed by a drastic increase of their absolute values (Figure 3a,b). This behavior was reproducible for films of different thickness (Supplementary Material Section S3). When applying the viscoelastic film in air model to interpret this data, it follows that it corresponds to drastic increase in the water content and viscous character of the coatings (Figure 3c,d).

#### 3.2.3. Water Sorption Isotherms of Rhodacal^®^ DSB Films

**Figure 4:**
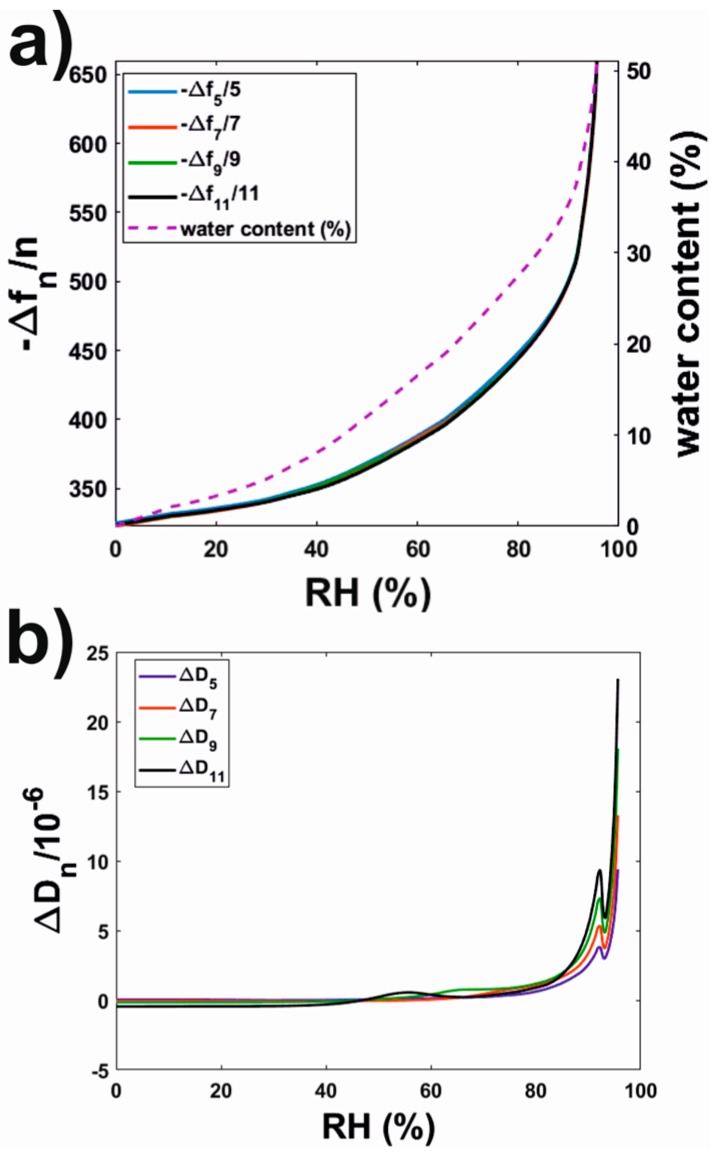
(**a**) Frequency and (**b**) dissipation shifts for the water sorption isotherm of a Rhodacal^®^ DSB spin-coated film.

Films of Rhodacal^®^ DSB were formed on clean QCM-D SiO_2_ sensors by means of the same procedure as for the formation of latex coatings. Their water sorption isotherms were also characterized in the same way (Figure 4).

In this case, Δ*f_n_*/*n* did not exhibit a dependence with the overtone number (Figure 4a), and Δ*D_n_* (Figure 4b) exhibited significantly lower values than those observed for the latex coating (Figure 3b). This indicates that Rhodacal^®^ DSB films exhibited an elastic character (i.e., solid-like). In this situation, the Sauerbrey model (Equation (1)) can be used to calculate the adsorbed mass. From this calculation, and then by applying Equation (4), we also calculated the water content of the Rhodacal^®^ DSB films (Figure 4a). Rhodacal^®^ DSB and latex films exhibited a somehow similar behavior. Up to a RH value of approximately 90% the absolute frequency shift values (and, therefore, the absorbed water) increased smoothly with RH for both systems. The similar trend observed for dissipation shifts indicated that this increase in water content led to softer films. Above this RH threshold, both signals showed a steeper dependence with RH. Moreover, dissipation shifts exhibited a similar peak/trough feature as the one reported above for the latex coatings.

### 3.3. Differential Scanning Calorimetry (DSC)

**Figure 5:**
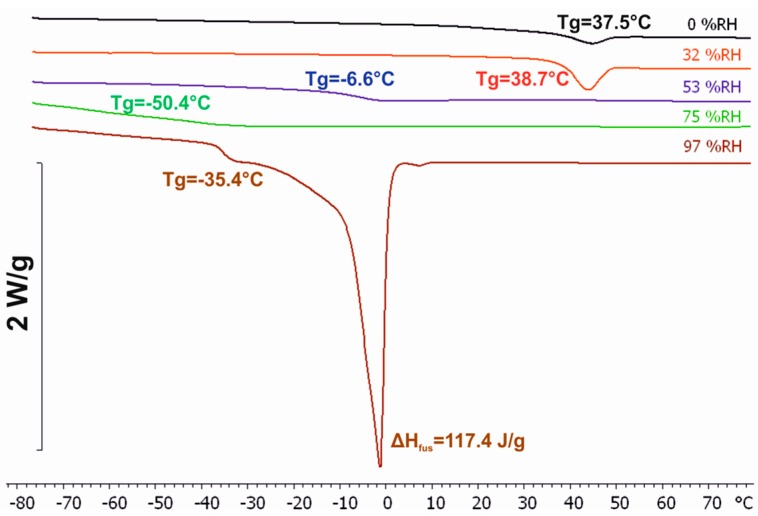
DSC scans of Rhodacal^®^ DSB equilibrated at different relative humidities (RHs, the specific value is provided above each scan).

Representative DSC scans of Rhodacal^®^ DSB samples equilibrated at different RH values are shown in Figure 5. Endothermic steps can be observed for all DSC scans. At first sight, it is not clear whether the steps observed for RHs 0% and 32% corresponded to glass or melting transitions. However, second consecutive scans of the samples confirmed they corresponded to a glass transition followed by a structural relaxation (Supplementary Material Section S4). All other scans revealed endothermic steps that could be unequivocally associated as well with glass transitions. *T*_g_ (midpoint) values for all DSC scans are also included in Figure 5. Overall, Figure 5 shows that when increasing the equilibration RH from 0% to 32% *T*_g_ did not significantly change. Further increase of RH led to a decrease of *T*_g_. This could be explained by plasticization effects [28]. However, *T*_g_ increased again for samples equilibrated at RH values (97% RH in Figure 5) higher than the threshold for which QCM-D revealed a RH-induced transition (~90% RH). Moreover, only at this RH (97%) did we observe the large endothermic peak characteristic of ice melting. The enthalpy of fusion associated with this peak was 117.4 J/g, indicating that Rhodacal^®^ DSB equilibrated at RH 97% contained ca. 35% of freezing water. Thus, DSC experiments support a scheme where Rhodacal^®^ DSB undergoes at these high RH values a phase transition (as indicated by the increase in *T*_g_) that its accompanied by a drastic increase of its ability to uptake free (freezing) water.

### 3.4. Atomic Force Microscopy (AFM)

**Figure 6:**
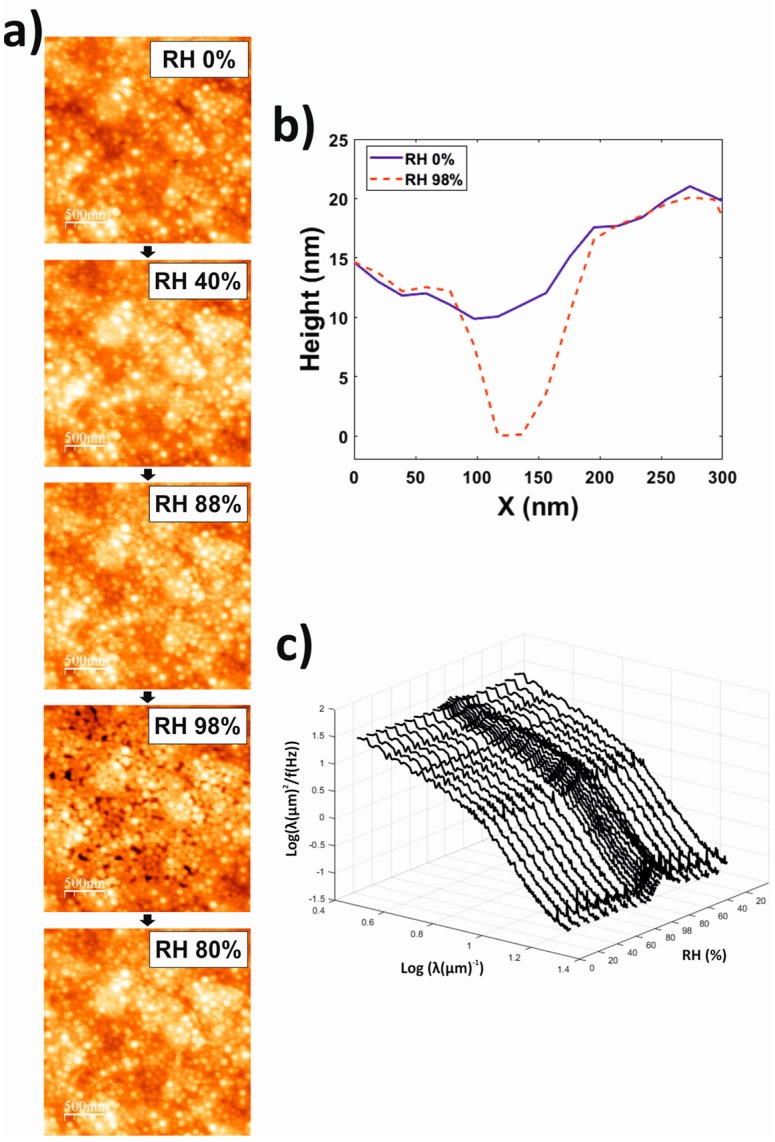
(**a**) Representative AFM topography images (height color scale 0–33 nm) of the latex coating obtained during a cycle where the RH was increased from 0% to 98% and then decreased. (**b**) Representative height profiles along the same lateral positions of the same latex coating, obtained at RH 0% and RH 98%., where a hole appeared at the highest RH. (**c**) Power Spectral Density plots calculated for the AFM topography images (10 µm × 10 µm, 512 × 512 points) obtained for the latex coating while varying RH. λ represents the roughness scale and f the scanning speed [29].

The topography of the latex coatings investigated by means of QCM-D was visualized afterwards with an AFM. In these experiments RH was controlled, and initially ramped from 0% to 98% and then down to 0%. Representative images at successive RH conditions are shown in Figure 6a.

For RH < 90%, AFM revealed a mostly coalesced film, even though some spherical features, which could be associated with noncoalesced polymer particles, were still observed. For RH > 90% i.e., RH conditions for which QCM-D signals exhibited peak/trough features followed by steeper RH-dependences in water sorption isotherm experiments (Figure 3a,b), AFM revealed that the surface of the coatings was populated by holes. These holes could achieved widths in the order of ca. 100 nm and depths of ca. 10 nm (Figure 6b). The formation of holes was a reversible process as indicated by their disappearance when RH was decreased afterwards. Classical roughness parameters such as the rms height did not clearly reflect these topographical changes. Instead, the RH-dependence of the sample topography could be clearly observed when plotting the PSD 1D roughness (calculated as in [29]) for the whole RH ramping process (Figure 6c). Indeed, the PSD roughness characterization could be used not only for the identification of the RH interval where the topographical changes occurred, but also to estimate the length scale of these changes. As observed in Figure 6c, the change in roughness can be clearly observed around a roughness scale (λ) of ~100 nm i.e., ca. the maximum width of the holes observed in the topography images.

**Figure 7:**
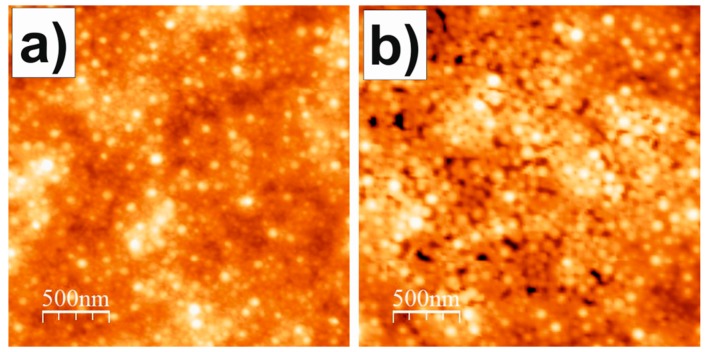
Topography of the latex coating at RH 0% obtained (**a**) before and (**b**) after water rinsing. Color height scale 0–33 nm.

The latex coatings were further characterized at 0% RH after being rinsed with UHQ water and subsequently N_2_-dried. Figure 7 shows AFM images of the latex coating before and after being rinsed with UHQ water (both obtained at 0% RH). It is clear that similar holes as those observed for the non-rinsed coatings at RH > 90% (Figure 6a) can also be observed at lower RH if the coatings were previously rinsed with water.

## 4. Discussion

One of the main functions of latex coatings is to protect the underlying substrates from mechanical insults. Additionally, in some applications e.g., wood finishing, it is of high importance that the coatings function as well as water diffusion barriers [30], a property that is directly linked to their water sorption properties. Both functions i.e., protection from mechanical insults and from water, are closely interconnected as a result of water plasticization effects [31]. This was indeed the starting point of this work, specifically our observation that the higher the equilibration RH the lower the hardness of the coatings (Table 1). This was determined by means of the König pendulum test. However, while this technique is widely used by the industry, it provides little mechanistic insight. To further investigate this observation we employed different techniques, specifically QCM-D, AFM and DSC where we implemented humidity control.

QCM-D is an optimal technique to provide further insight into the results provided by the König pendulum tests. Many different definitions can be found for the term “hardness” in the literature. The König pendulum provides the ability of a coating to damp the oscillating pendulum used as a probe. This is, the “hardness” provided by the König pendulum is indeed a (qualitative) characterization of the viscoelasticity of the coatings. As we have seen, QCM-D does not only provided information on the water content of the coatings but also on their viscoelastic character (in a quantitative way). Indeed, QCM-D has previously been used to characterize the mass and mechanical properties of latex coatings [32,33], even at different RH values [34]. In this work, we first employed QCM-D to investigate the film formation process. For this we focused on spin-coated films as this deposition method provided reproducible results. Of interest is that spin-coated films reached stable mass and viscoelasticity values after ca. 2 h (as monitored by QCM-D, Figure 1). This relatively short period was probably a consequence of the low thickness of the spin-coated films, which was in the order of 1.5 µm (assuming a mass of 1.5 g/m^2^ and a density of 10^3^ g/m^3^). Figure 1 reveals another interesting observation i.e., a change in the ambient humidity led to an almost simultaneous change in the mass and viscoelasticity of the latex coating. This was in accordance with König pendulum tests, which revealed that the hardness of coatings equilibrated at different RHs reached again similar values after being exposed to the same RH for ca. 5 min. This indicates that cured latex coatings rapidly respond to RH changes by absorbing/releasing water content, a process that has a significant influence in their mechanical properties (as expected from the plasticization effects of water).

We also showed that the model for viscoelastic films in air [24] could be used to fit QCM-D results obtained for latex coatings (Figure 2), allowing the estimation of the dependence of their water content and viscoelasticity (in terms of the *G*′/*G*″ ratio) at different RH values (Figure 2 and Figure 3). In this regard it is of relevance that the coatings exhibited a viscoelastic character even at 0% RH i.e., in the absence of water, suggesting that their structure was not homogeneous. AFM (10 µm × 10 µm) images of different zones of the samples did not reveal significant differences. Thus, structural in-homogeneities at a macroscopic scale can be discarded. However, AFM investigations revealed in-homogeneities at the microscale that could account for this viscoelastic character. First, AFM images (Figure 6a) evidenced that a non-negligible amount of the polymer particles contained in the original latex formulation did not coalesce during the film formation process. Moreover, AFM also revealed the presence of microdomains, probably formed by the surfactant used to stabilize the latex dispersion as discussed below, characterized by a dependence of their mechanical properties with RH that differed for that of the film matrix (Figure 6a).

In order to further explore the influence of RH on latex coatings we made full use of the QCM-D potential, and did not only use this technique to study the film formation process (Figure 1 and Figure 2), but also to continuously scan the RH-dependence of the water content and viscoelasticity of the investigated coatings (Figure 3). From the methodological point of view it is of relevance that this method, which was previously employed to characterize systems such as thin protein, lipid and surfactant films [20,21,35] can also be used to investigate significantly thicker polymer coatings. These QCM-D isotherms revealed that increasing the RH from 0% led initially to a continuous and smooth increase in the water content of the investigated coatings, and to a similarly smooth decrease of their *G*′/*G*″ ratio (i.e., to an increasing viscous character) which confirmed the plasticization effects of water. However, at a RH of ca. 90% the shape of Δ*f_n_*/*n* and Δ*D_n_* plots revealed peak/trough-like features followed at higher RHs by prominent and abrupt changes in these signals (Figure 3a,b). When modelled, these changes evidenced a steeper increase of the water content of the coatings accompanied by a similarly steeper decrease of their G′/*G*″ ratio (Figure 3c,d).

At this point, it is worth to note that the model used to fit QCM-D data (Equation (2)) is only strictly valid when the wavelength, λ, is higher than four times the film thickness, *d*_f_. When *d*_f_ ~ λ/4, the film forms a vibrating reed [24]. The thickness of the films investigated in this work are close to this limit. Thus, one should consider the scenario where increasing RH induced swelling of the films and that at high RH the films reached a thickness similar to the λ/4 limit, which could be the origin of the observed peak/trough-like features. However, the limit for the 5th overtone, λ_5_/4, is ca. twice as high as that for the 11th overtone, λ_11_/4, and in our experiments we observed that the peak/trough-like features were observed for similar RH for all overtones. Moreover, we observed that these features took place at similar RH for films of different thickness (Supplementary Material Section S3). Thus, it can be discarded that the peak/trough-like features observed both in Δ*f_n_*/*n* and Δ*D_n_*, which were followed by drastic changes in the mass and viscoelasticity of the films, were the result of the films entering in resonance with the vibrating substrate.

Similar experiments on films of well-characterized surfactants [35] revealed a similar behavior at RH values for which the surfactants underwent RH-induced transitions towards more hydrated and softer phases. This similarity led to the hypothesis of whether the observed behavior was originated by a phase transition of the surfactant used to stabilize the latex dispersion i.e., Rhodacal^®^ DSB (sodium alkyl diphenyl oxide sulfonate), which was expected to remain in the coatings in the form of domains. So far, a phase diagram has not been reported for this surfactant, even though its widespread use in the coating industry. Subsequently, QCM-D water sorption isotherms were also obtained for films of this surfactant (Figure 4). In this case, Δ*f_n_*/*n* was fairly independent of the overtone number, indicating the rigidity of the films. For RH values below 90%, −Δ*f_n_*/*n* (and, therefore, their mass/water content) increased smoothly with RH and Δ*D_n_* values remained fairly constant. However, for RH > 90% both −Δ*f_n_*/*n* and Δ*D_n_* increased drastically. Moreover, the drastic increase in Δ*D_n_* was preceded by peak/trough-like features similar to those observed for the latex coating. Such peak/trough-like features have been observed for other surfactants [35] and associated with the complex interactions between the two phases present during a phase transition. The occurrence of a phase transition was also supported by DSC investigations of Rhodacal^®^ DSB (Figure 5). DSC did not only reveal, for samples equilibrated at RH > 90%, an ice-melting endothermic peak but also a glass transition occurring at higher *T*_g_ than those observed for samples equilibrated at lower RHs. It is also of interest to discuss the enthalpy of fusion of the ice-meting endothermic peak. When compared to that of pure water, it revealed a content of free/freezing water for Rhodacal^®^ DSB equilibrated at RH 97% of ca. 35%. This is in accordance with QCM-D water sorption isotherms (Figure 4a) if we consider only the water absorbed from RH 90% (if extrapolated as in QCM-D experiments we did not reach RH 97%). Comparison of both results suggest that whereas Rhodacal^®^ DSB absorbs water continuously below RH 90%, this is in a bound/nonfreezing state and only above this threshold the surfactant contains free/freezing water (that revealed by DSC).

These similarities suggest that, at least at ambient temperature, Rhodacal^®^ DSB undergoes a transition towards a softer and more hydrated phase at ca. RH 90%. We do not have enough data though to infer which type of transition (we plan to investigate this aspect in the near future by means of X-ray scattering). Nevertheless, it follows from our data that the presence of this surfactant within the coatings, probably in the form of domains, is in the origin of the drastic decrease of the mechanical and water barrier properties at RH higher than 90%.

To further investigate the RH influence on the latex coatings, we visualized their topography by means of AFM while ramping the ambient RH (Figure 6a). When exposed to RH < 90%, the coatings exhibited a reasonable homogeneous topography. However, for RH > 90% the topography of the coatings was covered with holes of approximately 100 nm width and 10 nm depth. Interestingly, these holes disappeared once the RH was lowered again beyond this threshold value (Figure 6). For the interpretation of these results is worth to keep in mind that AFM does not provide the “true” topography, but isoforce maps instead i.e., the monitored topography corresponds to that for which the AFM cantilever applies a defined force value. Thus, our results indicate that the surface of the coatings presented different domains, one of them formed by a material which becomes significantly softer at RH > 90%. At these high RHs, when on top of these domains the separation between sample and the base of the AFM cantilever had to be lower than when on top of the rest of the surface in order to achieve a similar cantilever deflection i.e., the domains of the softer material appeared as holes. When RH was subsequently decreased below this threshold value, these domains became rigid again and they were no longer observed as holes. Interestingly, when the coatings were extensively rinsed with water and then dried, these holes were observed even for RH 0% (Figure 7). These experiments confirmed that the coating presented domains of a water-soluble material, and that this water-soluble material undergoes a transition at RH 90% towards a softer phase. Given that surfactants are the only water-soluble component of the coatings, and that the surfactant used to stabilize the latex dispersion that was employed to form the coatings i.e., Rhodacal^®^ DSB, also exhibited a transition towards a more hydrated and softer phase at RH 90%, it is reasonable to state that the domains that appeared as holes in the AFM images for RH > 90% were mainly composed of Rhodacal^®^ DSB. Overall, this further supports our interpretation of QCM-D data in that (i) the viscoelastic character of the coatings, at least at 0% RH, reflects a structural inhomogeneity and (ii) that the drastic decrease of the mechanical performance of the coatings observed for RH > 90% is a consequence of the presence of embedded surfactant domains that undergo a transition towards a more hydrated and softer phase at this RH threshold. At this point, it is worth to mention that QCM-D water sorption isotherms for coatings rinsed with water exhibited a similar behavior as those obtained for non-rinsed coatings. This indicates that Rhodacal^®^ DSB formed domains in the interior of the coatings and not only on their outer surface, as expected considering that QCM-D is a technique sensitive to the “bulk” of the coating. Regarding the decrease of the water barrier properties observed also for the RH 90% threshold, it is unlikely that the surfactant domains account for the entire water uptake occurring for RH > 90%. From AFM images it follows that the holes associated with surfactant domains occupy an area fraction of only ca. 2%. Probably their volume fraction within the whole coating is even lower given that surfactant tend to migrate to the coating interfaces [1]. Nevertheless, the fact is that the latex films exhibited a drastic increase of its ability to uptake water from the RH for which surfactant domains start to drastically increase their free/unbound liquid water content. This suggests that the presence of these liquid water reservoirs within the coating are in the origin of the decrease in the water barrier properties of the coatings as well.

Surfactants are often referred as a necessary evil in latex coatings. While their use is a must for stabilizing latex dispersions, their presence in the cured coatings often results in detrimental effects [1]. While there is vast literature on the presence, distribution and effects of surfactants in latex coatings, the fact is that there have been little advances on how to control these aspects (even though surfactant-free waterborne coatings can be achieved by means of more expensive chemistries e.g., by using waterborne polyurethanes). In this work, we bring attention into one effect of the presence of embedded surfactants in latex coatings that has not been previously reported to the best of our knowledge. Surfactants can exist in a variety of phases determined by different factors, among them environmental ones such as humidity. This is the case as well for the surfactant in focus in this work, Rhodacal^®^ DSB (sodium alkyl diphenyl oxide sulfonate), for which we report a transition towards a highly hydrated and soft phase at a RH of ca. 90%. We have shown that this transition, when occurring in domains of this surfactant embedded in latex coatings, leads to a drastic detriment of the water barrier and mechanical properties of the coatings. This observation can be easily extrapolated to the vast majority of latex coatings. Different humidities lead to different phases with different water sorption and mechanical properties for almost all type surfactants [35]. This effect is of high relevance for latex coatings as they are present in an extremely high number of different applications and, therefore, can be exposed to a wide range of different humidities. Thus, the characterization of the phase behavior of the surfactants used in latex dispersions would be of significant help in their rational and effective design as it would provide information critical for the prediction of their performance within the environmental conditions where they will be used, a long-term goal of the coating industry.

## 5. Conclusions

This work provides a solid scheme for the role of surfactant domains in latex coatings on their mechanical and water barrier properties. For this, we investigated a model latex coating at different RH values, still of industrial relevance, by means QCM-D, AFM and DSC. The use of QCM-D and AFM to study latex coatings is not new. However, this works proves the potential of controlling RH in these techniques. By means of this methodological approach, we showed that the surfactant used for stabilizing the latex dispersion forms domains within the resulting coatings. These coatings had a viscoelastic character even at 0% RH. In the absence of a plasticizing agent, the viscoelastic character would be the result of a structural inhomogeneity i.e., the presence of surfactant domains themselves. At higher humidity the water content and viscoelastic character of the coatings increased smoothly with RH, in a similar way as observed for films formed by the dispersion stabilizing surfactant i.e., Rhodacal^®^ DSB. This similarity, along with the hydrophobic character of the polymers of the coating, suggest that the uptake of water by the embedded surfactant domains could be ascribed as the mechanism responsible for the RH-induced plasticization of the coatings. Finally, this work highlights the drastic effect that phase transitions of the embedded surfactants can have on the properties of latex coatings. We showed that Rhodacal^®^ DSB, undergoes a transition towards a more hydrated and softer phase at a RH of ca. 90%. Exposing coatings formed from dispersions stabilized by this surfactant to RHs above this threshold value resulted in an abrupt decrease of its mechanical and water barrier properties. This result is also of relevance for other coatings formed from latex dispersions stabilized by different surfactants. It is well-known that most latex coatings contain embedded surfactant domains [1] and that RH induces similar phase transitions on almost all types of surfactants [35]. Thus, the presented results highlight the importance of considering the phase behavior of the surfactants used in latex dispersions during their design, as this will have a critical influence on their performance.

## Figures and Tables

**Table 1:** Hardness of coatings, as determined by the König pendulum test, formed at 50% RH for 1 week and then equilibrated at 50% RH and at 100% RH for 7 and 14 days.

Equilibration RH	# Oscillations (König Hardness) Equilibration Period 7 days	# Oscillations (König Hardness) Equilibration Period 14 days
50%	60.5 ± 0.7	63.5 ± 0.7
100%	43.0 ± 4.2	35.0 ± 7.1

## Supplementary Materials

The following are available online at http://www.mdpi.com/2073-4360/10/3/284/s1, Section S1: QCM-D characterization of the initial stages of the film formation process, Section S2: QCM-D half-band-half-width shifts monitored during the drying of the investigated latex dispersion, Section S3: Reproducibility of QCM-D water sorption isotherms, Section S4: Glass transition of Rhodacal^®^ DSB equilibrated at low RH values.
