# Supplementary material for: Humidity-Induced Phase Transitions of Surfactants Embedded in Latex Coatings Can Drastically Alter Their Water Barrier and Mechanical Properties
Source: Polymers (Basel). 2018 Mar 8;10(3):284. doi: 10.3390/polym10030284 (PMC6415026; doi:10.3390/polym10030284)
Supplement: Supplementary file 1 [file polymers-10-00284-s001.pdf]

Humidity-induced phase transitions of surfactants embedded in latex coatings can drastically alter their water barrier and mechanical properties

- Supplementary Material –

Juan F. Gonzalez-Martinez [1], Yana Znamenskaya Falk [1], Sebastian Björklund [1], Stefan Erkselius [2], Nicola Rehnberg [2] and Javier Sotres [1]

[1] Biomedical Science Department & Biofilms-Research Center for Biointerfaces, Malmö University, 20506 Malmö, Sweden

[2] Bona AB, 20021 Malmö, Sweden;

S1. QCM-D characterization of the initial stages of the film formation process

As mentioned in the main manuscript, QCM-D monitoring of the film formation process revealed that both frequency and dissipation signals were stabilized after the first 2 hours. This is evidenced in the plot below (Figure S1) where the frequency and dissipation signals monitored for the first 2 hours of the drying of a representative latex dispersion are shown:

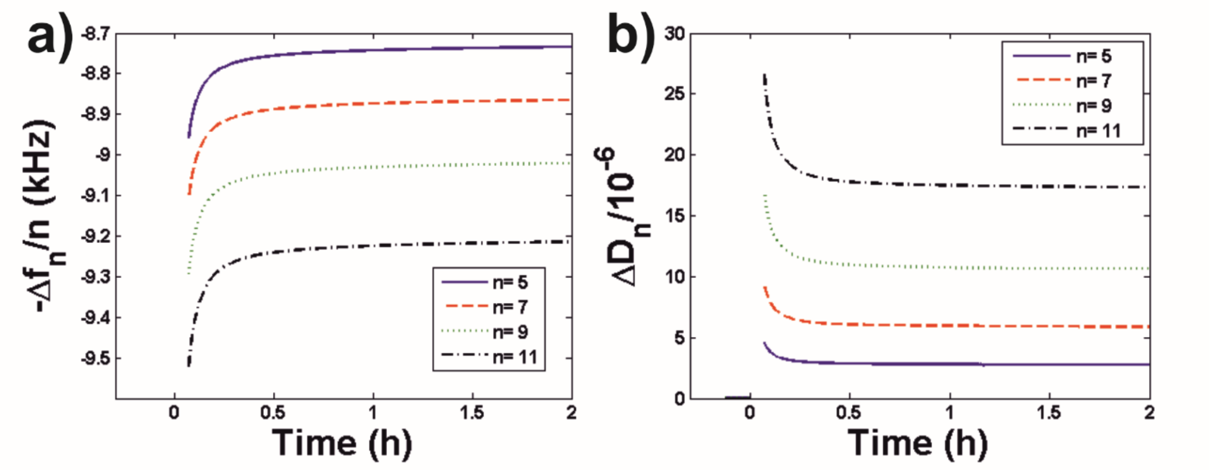

Figure S1. a) Frequency and **b)** dissipation QCM-D shifts monitored during the first 2 hours of the drying of the investigated latex dispersion.

S2. QCM-D half-band-half-width shifts monitored during the drying of the investigated latex dispersion

The model used for determining the G′/G′′ ratio of the investigated coatings in the manuscript (Eq. 3) makes use of half-band-half-width shifts, $\Delta\Gamma_n$, where $\Gamma_n = f_n D_n/2$. In order to illustrate the behaviour of this quantity, its value calculated from the frequancy and dissipation shifts shown in Figure 1 in the manuscript (corresponding to the film formation process) is shown below:

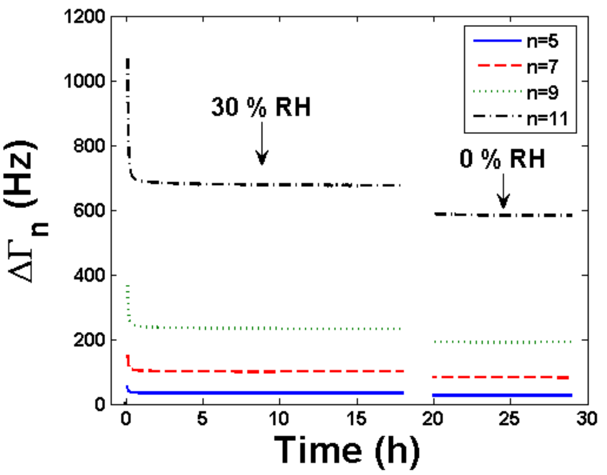

Figure S2. QCM-D half-band-half-width shifts monitored during the drying of the investigated latex dispersion.

S3. Reproducibility of QCM-D water sorption isotherms

The QCM-D results reported in the main manuscript, and specifically the peak/trough-like features of $\Delta f_n/n$ and ΔD_n at a RH of ca. 90%, were highly reproducible even for films of different thickness. This is shown in Figure S1, which represents shifts in the frequency and dissipation signals of the 7th overtone monitored for different latex coatings during water sorption experiments.

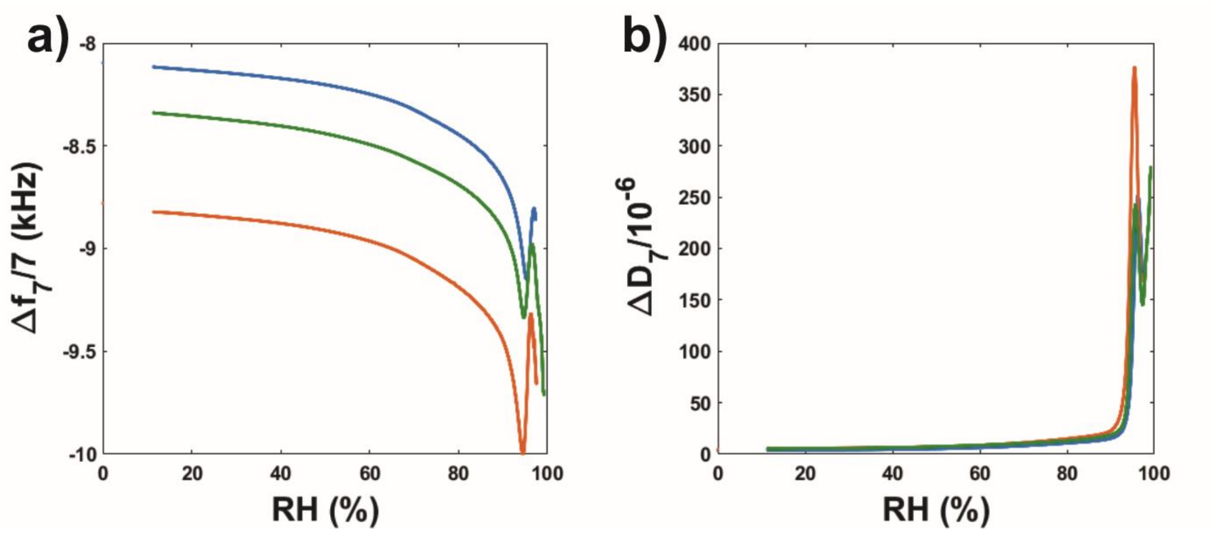

Figure S1. a) Frequency and **b)** dissipation shifts corresponding to the 7th overtone of different latex coatings monitored during water sorption experiments.

S4. Glass transition of Rhodacal ® DSB equilibrated at low RH values

For DSC experiments, Rhodacal ® DSB samples were equilibrated at different RH, but always at room temperature (ca. 25 ˚C). When a glassy material is stored below its glass transition temperature, it can spontaneously approach a more stable state (Liu et al., 2006). This phenomenon is called enthalpy relaxation. At this point, increasing the temperature above T_g results in structural relaxations that in DSC scans are observed as enthalpy recovery peaks above and below the glass transition region (Liu et al., 2006; Fan et al., 2016). This can make it difficult to discern in DSC scans if the transition is indeed associated with a glass transition. A way to approach this problem is to perform a second DSC scan immediately after the first one i.e., not allowing any aging related enthalpy relaxation. If an endothermic step is associated with a glass transition, enthalpy recovery peaks should not be observed in the second scan (Liu et al., 2006; Fan et al., 2016).

We observed such transitions for Rhodacal ® DSB samples equilibrated at low RH (Figure 5 in the manuscript). In these cases (RHs 0% and 32%), endothermic steps were observed at temperatures above the room temperature i.e., the temperature during equilibration of the samples. However, in second scans enthalpy recovery peaks were not observed (Figure S4), confirming that the observed endothermic steps could be unequivocally associated with glass transitions.

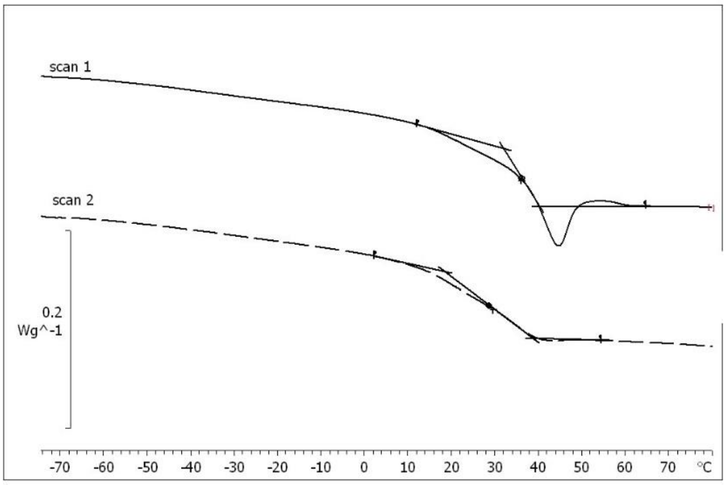

Figure S4. Consecutive DSC scans of Rhodacal ® DSB equilibrated at RH 0%.

Fan, F.; Roos, Y.H. Structural relaxations of amorphous lactose and lactose-whey protein mixtures. J. Food Eng., 2016. 173, 106-115.

Liu, Y; Bhandari B.; Zhou, W. Glass transitions and enthalphy relaxation of amorphous food saccharides: a review. J. Agric. Food Chem., 2006. 54, 5701-5717.
